# Efficacy and Safety of Ibrutinib Therapy in Patients with Chronic Lymphocytic Leukemia: Retrospective Analysis of Real-Life Data

**DOI:** 10.4274/tjh.galenos.2021.2021.0007

**Published:** 2021-12-07

**Authors:** Anıl Tombak, Funda Pepedil Tanrıkulu, Salih Sertaç Durusoy, Hüseyin Derya Dinçyürek, Emin Kaya, Elif Gülsüm Ümit, İrfan Yavaşoğlu, Özgür Mehtap, Burak Deveci, Mehmet Ali Özcan, Hatice Terzi, Müfide Okay, Nilgün Sayınalp, Mehmet Yılmaz, Vahap Okan, Alperen Kızıklı, Ömer Özcan, Güven Çetin, Sinan Demircioğlu, İsmet Aydoğdu, Güray Saydam, Eren Arslan Davulcu, Gül İlhan, Mehmet Ali Uçar, Gülsüm Özet, Seval Akpınar, Burhan Turgut, İlhami Berber, Erdal Kurtoğlu, Mehmet Sönmez, Derya Selim Batur, Rahşan Yıldırım, Vildan Özkocamaz, Ahmet Kürşad Güneş, Birsen Sahip, Şehmus Ertop, Olga Meltem Akay, Abdülkadir Baştürk, Mehmet Hilmi Doğu, Aydan Akdeniz, Ali Ünal, Ahmet Seyhanlı, Emel Gürkan, Demet Çekdemir, Burhan Ferhanoğlu

**Affiliations:** 1 Mersin University Faculty of Medicine, Department of Internal Medicine, Division of Hematology, Mersin, Turkey; 2 Başkent University Adana Application and Research Center, Adana, Turkey; 3 Gaziantep University Faculty of Medicine, Department of Hematology, Gaziantep, Turkey; 4 Çukurova University Faculty of Medicine, Department of Hematology, Adana, Turkey; 5 İnönü University Turgut Özal Medical Center, Department of Hematology, Malatya, Turkey; 6 Trakya University Faculty of Medicine, Department of Hematology, Edirne, Turkey; 7 Adnan Menderes Univercity Faculty of Medicine, Department of Hematology, Aydın, Turkey; 8 Kocaeli University Faculty of Medicine,Department of Hematology, Kocaeli, Turkey; 9 Medstar Antalya Hospital, Clinic of Hematology, Antalya, Turkey; 10 Dokuz Eylül University Faculty of Medicine, Department of Hematology, İzmir, Turkey; 11 Cumhuriyet University Faculty of Medicine, Department of Hematology, Sivas, Turkey; 12 Hacettepe University Faculty of Medicine, Department of Internal Medicine, Division of Hematology, Ankara, Turkey; 13 Bezmialem Vakıf University Faculty of Medicine, Department of Hematology, İstanbul, Turkey; 14 Necmettin Erbakan University Meram Faculty of Medicine, Department of Hematology, Konya, Turkey; 15 Celal Bayar University Faculty of Medicine, Department of Hematology, Manisa, Turkey; 16 Ege University Hospital, Clinic of Internal Medicine, Division of Hematology İzmir, Turkey; 17 Mustafa Kemal University Faculty of Medicine, Department of Internal Medicine, Hatay, Turkey; 18 Ankara Numune Training and Research Hospital, Clinic of Hematology, Ankara, Turkey; 19 Namık Kemal University Faculty of Medicine, Department of Hematology, Tekirdağ, Turkey; 20 Antalya Training and Research Hospital, Clinic of Hematology, Antalya, Turkey; 21 Karadeniz Technical University Faculty of Medicine, Department of Hematology, Trabzon, Turkey; 22 Ataturk University Faculty of Medicine, Department of Hematology, Erzurum, Turkey; 23 Uludağ University Faculty of Medicine, Division of Hematology, Bursa, Turkey; 24 Şanlıurfa Mehmet Akif İnan Training and Research Hospital, Clinic of Hematology, Şanlıurfa, Turkey; 25 Zonguldak Bülent Ecevit University Faculty of Medicine, Department of Hematology, Zonguldak, Turkey; 26 Koç University Faculty of Medicine, Department of Hematology, İstanbul, Turkey; 27 Konya Training and Research Hospital, Clinic of Internal Medicine, Konya, Turkey; 28 İstanbul Training and Research Hospital, Clinic of Hematology, İstanbul, Turkey; 29 Erciyes University Faculty of Medicine, Department of Internal Medicine, Kayseri, Turkey; 30 Ege University Faculty of Medicine, Department of Hematology, İzmir, Turkey; 31 Anadolu Medical Center, Bone Marrow Transplantation Center, Department of Hematology, Kocaeli, Turkey; 32 İstanbul University-Cerrahpaşa Cerrahpaşa Faculty of Medicine, Department of Internal Medicine Section of Haematology, İstanbul, Turkey

**Keywords:** Chronic lymphocytic leukemia, Ibrutinib, Bruton’s tyrosine kinase inhibitor

## Abstract

**Objective:**

This study aimed to retrospectively evaluate the efficacy, safety, and survival outcome of single-agent ibrutinib therapy in chronic lymphocytic leukemia patients.

**Materials and Methods:**

A total of 136 patients (mean age ± standard deviation: 64.6±10.3 years, 66.9% males) who had received at least one dose of ibrutinib were included in this retrospective multicenter, noninterventional hospital-registry study conducted at 33 centers across Turkey. Data on patient demographics, baseline characteristics, laboratory findings, and leukemia-cell cytogenetics were retrieved. Treatment response, survival outcome including overall survival (OS) and progression-free survival (PFS), and safety data were analyzed.

**Results:**

Overall, 36.7% of patients were categorized as Eastern Cooperative Oncology Group (ECOG) class 2-3, while 44.9% were in Rai stage 4. Fluorescence in situ hybridization revealed the presence of del(17p) in 39.8% of the patients. Patients received a median of 2.0 (range: 0-7) lines of pre-ibrutinib therapy. Median duration of therapy was 8.8 months (range: 0.4-58.0 months). The 1-year PFS and OS rates were 82.2% and 84.6%, respectively, while median PFS time was 30.0 (standard error, 95% confidence interval: 5.1, 20.0-40.0) months and median OS time was 37.9 (3.2, 31.5-44.2) months. Treatment response (complete or partial response), PFS time, and OS time were better with 0-2 lines versus 3-7 lines of prior therapy (p<0.001, p=0.001, and p<0.001, respectively), with ECOG class 0-1 versus class 2-3 (p=0.006, p=0.011, and p=0.001, respectively), and with Rai stage 0-2 versus 3-4 (p=0.002, p=0.001, and p=0.002, respectively). No significant difference was noted in treatment response rates or survival outcome with respect to the presence of comorbidity, bulky disease, or del(17p). While 176 adverse events (AEs) were reported in 74 (54.4%) patients, 46 of those 176 AEs were grade 3-4, including pneumonia (n=12), neutropenia (n=11), anemia (n=5), thrombocytopenia (n=5), and fever (n=5).

**Conclusion:**

This real-life analysis confirms the favorable efficacy and safety profile of long-term ibrutinib treatment while emphasizing the potential adverse impacts of poorer ECOG performance status, heavy treatment prior to ibrutinib, and advanced Rai stage on patient compliance, treatment response, and survival outcomes.

## Abstract (tr)

**Amaç:**

Kronik lenfositik lösemi hastalarında tek ajan ibrutinib tedavisinin etkinliğini, güvenliğini ve sağkalım sonuçlarını geriye dönük olarak değerlendirmek.

**Gereç ve Yöntemler:**

Otuz üç merkezde yapılan bu retrospektif, çok merkezli, girişimsel olmayan hastane kayıt çalışmasına en az bir doz ibrutinib uygulanan 136 hasta (ortalama ± standart sapma yaş 64,6 10,3, % 66,9’u erkek) dahil edildi. Hastaların demografik verileri, bazal karakteristikleri, laboratuvar bulguları, lösemi hücre sitogenetiği ile ilgili veriler kaydedildi. Tedavi yanıtı, genel sağkalım (OS), progresyonsuz sağkalım (PFS) ve güvenlik verileri analiz edildi.

**Bulgular:**

Hastaların %36,7’sinde ECOG 2-3, % 44,9’u Rai evre 4 idi. FISH ile hastaların %39,8’inde del(17p) varlığını gösterdi. Hastalar medyan 2 (0 ila 7 arasında) sıra pre-ibrutinib tedavisi aldı. Medyan tedavi süresi 8,8 aydı (0,4-58 ay). Bir yıllık PFS ve OS oranları sırasıyla %82,2 ve %84,6, medyan (SE, %95 güven aralığı) PFS süresi 30 (5,1, 20-40) ay ve OS süresi 37,9 (3,2, 31,5-44,2) aydı. Tedavi yanıtı (CR veya PR), PFS ve OS süreleri; ibrutinib öncesi 3-7 basamak tedaviye karşı 0-2 basamak tedavi alanlarda (p<0,001, p=0,001 ve p<0,001, sırayla), ECOG 2-3’e göre ECOG 0-2 olanlarda (p=0,006, p=0,011 ve p=0,001, sırasıyla), Rai evre 0-2 olanlarda Rai evre 3-4 olanlara göre (p=0,002, p=0,001 and p=0,002, sırasıyla) daha iyiydi. Komorbidite, hacimli hastalık veya del(17p) varlığına göre tedaviye yanıt oranlarında veya sağkalım sonuçlarında önemli bir fark kaydedilmedi. 74 hastada (%54,4) 176 advers olay (AE) saptandı; 176 AE’nin 46’sı derece 3-4 idi. Bunlar; pnömoni (n=12), nötropeni (n=11), anemi (n=5), trombositopeni (n=5) ve ateş (n=5) idi.

**Sonuç:**

Bu gerçek hayat analizi, uzun vadeli ibrutinib tedavisinin olumlu etkililiğini ve güvenlik profilini doğrularken, kötü ECOG performans durumunun, ibrutinib’den önce ağır şekilde tedavi verilmiş olmasının ve ileri evre hastalığın, hasta uyumu, tedavi yanıtı ve sağkalım üzerindeki potansiyel olumsuz etkilerini ortaya koymuştur.

## Introduction

Owing to novel therapeutics such as combination chemotherapy with fludarabine and cyclophosphamide (FC) and chemoimmunotherapy with rituximab (FCR), the survival outcome and long-term remission rates of chronic lymphocytic leukemia (CLL) patients have improved significantly over the last decade, particularly in younger, low-risk CLL patients [1,2,3,4,5]. However, older patients with higher-risk genetic abnormalities or del(17p) still have inferior survival outcomes, while significant toxicities of chemotherapeutic regimens and poor survival rates with the use of conventional salvage regimens following relapse after FCR are also considered challenging factors in the management of CLL [3,4,6,7,8].

Given the importance of B-cell-receptor signaling in CLL and the central role of Bruton’s tyrosine kinase (BTK) in this pathway, targeted therapy with kinase inhibitors has become an alternative to conventional therapy for CLL [9,10,11]. The introduction of ibrutinib, an irreversible inhibitor of BTK, enabled significant improvement in the survival outcomes of CLL patients [10,11]. The results from three phase III trials demonstrated improved progression-free survival (PFS) and overall survival (OS) with ibrutinib compared to FCR or chlorambucil [12,13,14], while data from the RESONATE trial indicated the association of ibrutinib with significantly improved PFS, OS, and overall response rate (ORR) when compared to ofatumumab in previously treated CLL patients with several high-risk prognostic factors [15]. Accordingly, ibrutinib has become the standard of care in relapsed/refractory patients and is now being recommended for use in front-line treatment of patients regardless of age or del(17p) status [16,17,18,19,20,21].

Given the potential differences in baseline characteristics and treatment responses of patients recruited in clinical trials and those treated outside of clinical trials, there is considerable interest in real-world experience with the use of novel targeted drugs in the management of CLL patients, particularly for drugs such as ibrutinib that are recommended to be used continuously until progression [10,22,23,24,25]. This real-life multicenter study was therefore designed to retrospectively evaluate efficacy and safety along with survival outcomes of single-agent ibrutinib therapy in CLL patients who were treated outside the setting of clinical trials.

## Materials and Methods

### Study Population

A total of 136 adult patients diagnosed with CLL (≥18 years old; mean age ± standard deviation: 64.6±10.3 years; 66.9% male patients) who had received at least one dose of single-agent ibrutinib therapy after January 2013 were included in this retrospective multicenter, noninterventional hospital-registry study conducted between December 2018 and March 2019 at 33 centers across Turkey. Patients who had sensitivity to an active ingredient or component of the medication or who had ibrutinib treatment before December 2012 were excluded.

The study was conducted in full accordance with local good clinical practice guidelines and current legislations, while permission was obtained from the relevant institutional ethics committee for the use of patient data for publication purposes.

### Data Collection

Data on patient demographics (age, gender), baseline characteristics (comorbidity, bulky disease, organomegaly, infection, Eastern Cooperative Oncology Group [ECOG] performance status, Rai stage, previous treatments), and laboratory findings including hemoglobin, platelet count, leukocyte count, lymphocyte count, erythrocyte sedimentation rate, lactate dehydrogenase level, beta-2 microglobulin and IgG levels, Coombs test, and leukemia-cell cytogenetics (metaphase karyotyping, interphase fluorescence in situ hybridization [FISH] analysis) were retrieved from hospital records. Treatment responses including partial response (PR), complete response (CR), stable disease (SD), and progressive disease as well as final treatment response (PR and CR) were evaluated according to the relevant International Workshop Group on CLL response criteria [25]. Assessment of response was performed at least 2 months after achieving “maximum response”. The OS (duration, rate), PFS (duration, rate), and adverse events (AEs) were also analyzed for patients who received single-agent ibrutinib treatment within the study period. PFS was defined as the period from the date of ibrutinib initiation to the first recurrence/death or the last follow-up. OS was defined as the period from the date of diagnosis to death or last follow-up.

### Statistical Analysis

Statistical analysis was conducted using IBM SPSS Statistics 22.0 for Windows (IBM Corp., Armonk, NY, USA). Descriptive statistics were used to summarize baseline characteristics. Pearson’s chi-square (χ^2^) test was used for the comparison of categorical data. Survival analysis was performed via Kaplan-Meier analysis and comparisons were made via log-rank test. Data were expressed as mean ± standard deviation, median (minimum-maximum), 95% confidence interval (CI), and/or percentage (%) as appropriate.

## Results

### Baseline Characteristics

The mean patient age was 64.6±10.3 (range: 39-94) years and 61.9% of patients were male. Diabetes mellitus (25.7%) and hypertension (22.9%) were the most common comorbidities, while hepatosplenomegaly was noted in 33.8% of patients. Overall, 36.7% of patients were categorized as ECOG performance status class 2-3 and 44.9% were in Rai stage 4 (44.9%), while FISH testing revealed the presence of del(17p) in 39.8% of the patients (Table 1).

### Prior Lines of Therapy and Related Treatment Responses

Patients received a median of 2.0 (range: 0-7) lines of pre-ibrutinib therapy. CR rates were 27.8%, 32.8%, 10.7%, and 15.4% for patients having received 1, 2, 3, and ≥4 lines of prior therapy (Table 2).

### Characteristics of Ibrutinib Therapy

For the majority of patients, ibrutinib was administered orally at a daily dose of 420 mg. The treatment indications were B signs and stage 4 disease in 52.2% and 41.2% of patients, respectively (Table 3).

Median duration of ibrutinib therapy was 8.8 months (range: 0.4-58.0 months), while dose reduction, dose delay, treatment discontinuation, and AEs occurred in 16.9%, 26.5%, 24.3%, and 54.4% of patients, respectively (Table 3).

Lymphocyte counts increased within the first month of treatment, followed by a gradual decrease starting from the second month and resolving at the sixth month (Table 3).

### Treatment Response and Survival Outcome with Respect To Prognostic Factors

Final treatment response (CR or PR) was better in patients with 0-2 lines versus 3-7 lines of prior therapy (79.3% vs. 41.5%, p<0.001), in patients with ECOG performance status class 0-1 versus class 2-3 (75.0% vs. 50.0%, p=0.006), and in patients with Rai stage 0-2 versus 3-4 (88.9% vs. 57.0%, p=0.002). No significant difference was noted in final treatment response rates with respect to presence of comorbidity, bulky disease, or del(17p) status (Table 4).

After a median of 69.0 (range: 9.0-296.0) months of follow-up, mortality had occurred for 29 of 136 patients (21.3%), while 107 (81.3%) patients survived. Sepsis (31.0%) was the most common cause of death, followed by cardiac arrest (13.8%), pneumonia (10.3%), and Richter’s syndrome (10.3%) (Table 5).

**Figure 1:**
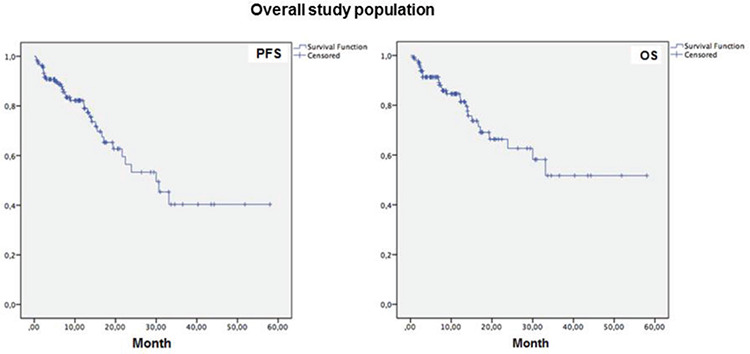
Overall 1-year progression-free survival (PFS) and overall survival (OS) rates.

Overall, 1-year PFS and OS rates were 82.2% and 84.6%, respectively (Table 5), while median (standard error [SE], 95% CI) PFS time was 30.0 (5.1, 20.0-40.0) months and median (SE, 95% CI) OS time was 37.9 (3.2, 31.5-44.2) months (Table 6, Figure 1).

**Figure 2:**
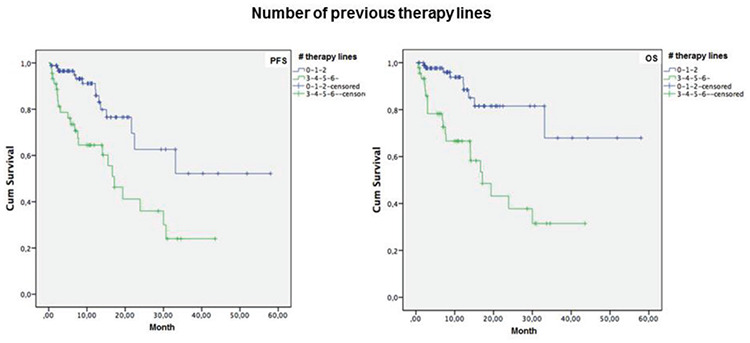
One-year progression-free survival (PFS) and overall survival (OS) rates in patients with 0-2 lines versus 3-7 lines of prior therapy.

**Figure 3:**
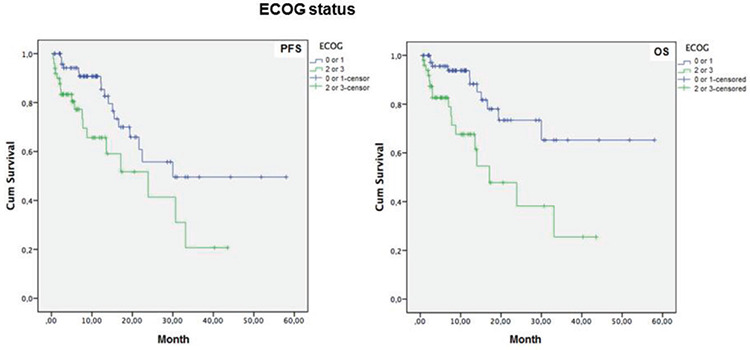
One-year progression-free survival (PFS) and overall survival (OS) rates in patients with Eastern Cooperative Oncology Group (ECOG) performance status class 0-1 versus class 2-3.

**Figure 4:**
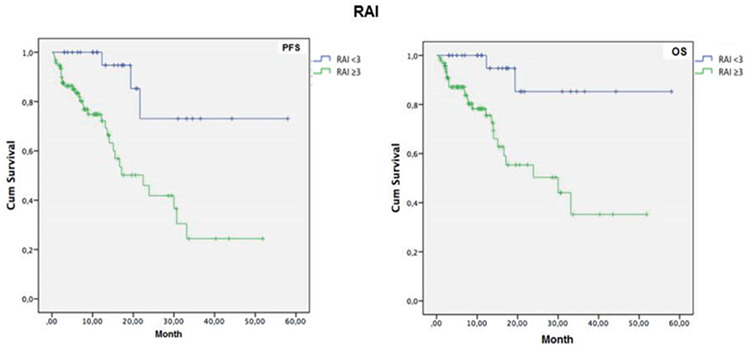
One-year progression-free survival (PFS) and overall survival (OS) rates in patients with Rai grade 0-2 versus 3-4.

Mean PFS time was longer in patients with 0-2 lines versus 3-7 lines of prior therapy (39.2±4.4 vs. 20.5±2.9 months, log-rank p=0.001, Figure 2), in patients with ECOG performance status class 0-1 versus class 2-3 (37.0±4.0 vs. 21.7±3.3 months, log-rank p=0.011, Figure 3), and in patients with Rai grade 0-2 versus 3-4 (47.5±5.4 vs. 24.7±3.0 months, log-rank p=0.001, Figure 4) (Table 6).

Mean OS time was also longer in patients with 0-2 lines versus 3-7 lines of prior therapy (45.9±4.19 vs. 22.1±3.1 months, log-rank p<0.001, Figure 2), in patients with ECOG performance status class 0-1 versus class 2-3 (43.7±3.9 vs. 22.1±3.49 months, log-rank p=0.001, Figure 3), and in patients with Rai stage 0-2 versus 3-4 (52.0±4.1 vs. 28.6±3.4 months, log-rank p=0.002, Figure 4) (Table 6).

No significant difference was noted in PFS time and OS time with respect to presence of comorbidity, bulky disease, del(17p) status, or overall FISH findings (Table 6).

### Safety Profile

Overall, 176 AEs were reported in 74 (54.4%) patients, and 46 of those 176 AEs were grade 3-4 AEs, including pneumonia (n=12), neutropenia (n=11), anemia (n=5), thrombocytopenia (n=5), and fever (n=5) in most cases. The atrial fibrillation rate was low (n=2) (Table 7).

## Discussion

Our findings revealed the favorable efficacy and safety profile of ibrutinib in CLL patients (mean age of 64.6 years, del(17p) mutation in 28.7%, Rai stage 3/4 in 68.4%) with 1-year PFS and OS rates of 82.2% and 84.6% at a median follow-up of 69.0 months, respectively. The final treatment response (CR or PR) was better and survival times (PFS and OS) were longer for patients with fewer than <2 lines of prior therapy, ECOG performance class 0-1, and Rai stage 0-2 while there was no significant impact of comorbidity, bulky disease, or del(17p) status on treatment response or survival outcomes.

Data from a real-life retrospective study including 32 ibrutinib-treated patients (11 had CLL) in Turkey revealed that in patients with CLL, ibrutinib treatment (median: 4 months) was associated with an ORR of 85.6% (28.5% CR and 57.1% PR) and occurrence of diarrhea in 3 (27.3%), pneumonia in 3 (27.3%), and thrombocytopenia and/or neutropenia in 2 (18.2%) patients [26]. The authors considered ibrutinib a good treatment option for CLL and other B-cell lymphomas, with an acceptable side-effect profile and a high and promising CR/PR response rate [26].

Similarly, according to real-life data from the UK CLL Forum obtained from 315 CLL patients with a median of 16 months of follow-up, the authors noted 1-year discontinuation-free survival (DFS) of 73.7% and 1-year OS of 83.8% with no significant difference in DFS and OS rates with respect to del(17p) status, whereas there was an association of better pre-treatment performance status (0/1 vs. 2+) with superior DFS (77.5% vs. 61.3%) and OS (86.3% vs. 76.0%) and an association of 1 prior line of therapy versus 2+ prior lines of therapy with a significant 1-year PFS advantage (94% vs. 82%) [22]. The same authors also noted no significant difference between more or less heavily pre-treated patients in terms of prognostic factors such as performance status and del(17p), while emphasizing the likelihood of older patients and those with del(17p) to have inferior DFS and OS when treated with ibrutinib beyond the second line [22].

In a multicenter Swedish study providing real-life data from 95 CLL patients (median age: 69 years, del(17p)/*TP53* mutation in 63%, Rai stage 3/4 in 65%), the authors reported that once-a-day ibrutinib treatment was well tolerated and associated with an ORR of 84%, PFS of 77%, and OS rate of 83% at a median follow-up of 10.2 months [23]. However, in contrast to our findings, the authors indicated that del(17p)/*TP53* mutation remained a therapeutic challenge given the significantly shorter PFS and OS in patients with del(17p)/*TP53* mutation [23].

In addition, data from a mutation analysis study of 63 patients who were still on ibrutinib after 3 years in an early-access program at 29 French centers revealed detection of *BTK* and *PLCG2* mutations in 57% and 13% of the next-generation sequencing samples (n=30) and the authors reported that after a median follow-up of 8.5 months from sample collection, the presence versus the lack of a BTK mutation was significantly associated with subsequent CLL progression [27]. The same authors emphasized a need for clinical trials to evaluate whether patients with BTK mutation may benefit from an early switch to another treatment [27].

In a real-life study on the efficacy of ibrutinib as a single agent in 180 patients with CLL recruited from three independent cohorts from Italy, 73 patients were reported to have discontinued ibrutinib for progression or for AEs, while *NOTCH1*-mutated patients were reported to have less redistribution lymphocytosis at 3 months on ibrutinib, to show inferior nodal response at 6 months, and to have significantly shorter PFS and OS [28]. The same authors noted that *NOTCH1* *M* plus lower BAX/BCL-2 ratio identified a CLL subset showing the worst PFS and OS, emphasizing the likelihood of either new small-molecule combination approaches or antibodies targeting *NOTCH1* being more appropriate therapeutic options for NOTCH1-mutated patients [28].

Notably, based on data from a study conducted in Poland on the potential significance of the mutational status of 30 selected genes for disease outcome in a real-life cohort of 45 heavily pretreated patients with CLL, the authors reported that despite the accumulation of several poor prognostic factors such as *TP53* (40.0%), *NOTCH1* (28.8%), *SF3B1* (24.4%), *ATM* (15.6%), *MED12* (13.3%), *CHD2* (11.1%), *XPO1* (11.1%), *NFKBIE* (11.1%), *BIRC3* (8.9%), *SPEN* (8.9%), *POT1* (8.9%), *EGR2 *(6.7%), and *RPS15* (6.7%) in their cohort, ibrutinib treatment showed long-term clinical benefits in terms of 36-month PFS (64.0%) and OS (68.2%) rates and the ORR (51.1%) [29].

Higher treatment response and better PFS and OS outcomes in patients previously treated with 0-2 lines of therapy versus more heavily treated patients in the current study seem to be consistent with data from other real-life studies [22]. Fewer lines of prior therapy were also reported to be associated with significantly improved PFS and OS outcomes and higher CR rates and 5-year PFS and OS rates in treatment-naive (TN) patients compared to relapsed/refractory (R/R) patients, emphasizing the deepening of responses with continued ibrutinib therapy and the likelihood of superior efficacy of initiating ibrutinib in earlier lines of therapy [16].

Dose reduction (16.9%), dose delay (26.5%), and treatment discontinuation (24.3%) rates in the current study also seem to be consistent with previous real-life data on ibrutinib discontinuation rates (10.5% to 17.5%), dose reductions (26.0%), and temporary treatment breaks (>14 days, 13.0%) or permanent treatment discontinuation (17.5% to 41%) [22,23,30,31]. Notably, neither the dose reductions nor the temporary treatment breaks were reported to be associated with survival outcome, whereas permanent cessation of ibrutinib was associated with reduced 1-year OS survival [22]. Similar to our findings, poorer 1-year DFS (16.2%) and OS (9.3%) in patients with poorer pre-treatment performance status (PS 2+) were reported while also noting a higher likelihood of treatment breaks within the first year of therapy in the PS 2+ group [22].

In a recent FILO Group study on the OS benefits of symptom monitoring in real-world CLL patients treated with ibrutinib, the authors reported that drug intolerance and toxicities (26.3%) rather than progressive disease accounted for most drug withdrawals [27] and they indicated the higher likelihood of stopping ibrutinib due to toxicities in the real-life setting when compared to ibrutinib discontinuation rates due to toxicity (10%) and CLL progression (13.5%) as reported in RESONATE and RESONATE-2 pooled analysis [32]. The potential role of certain factors in this discrepancy has been suggested, such as the clinical experience of physicians in managing toxicity, the availability of alternative therapy, and the characteristics of real-life populations in terms of performance status and comorbidities [31].

In a recent French study on patterns of use and safety of ibrutinib in real-life practice in 102 patients, half of whom were CLL patients, the authors reported that 42.1% of patients permanently discontinued ibrutinib in the first year, mostly for progression (51.2%) or adverse drug reactions (ADRs) (32.6%), while 47.1% of patients experienced at least one ibrutinib-associated serious ADR (SADR; hematological, infectious, and vascular disorders in particular) [33]. These authors also reported the probability of developing an ibrutinib-associated SADR to be 35.1% (95% CI: 26.3-45.7) at 3 months, 44.8% (95% CI: 35.2-55.8) at 6 months, and 54.3% (95% CI: 44.0-65.2) at 12 months, further indicating a significant association of age of ≥80 years (hazard ratio [HR]: 2.03; 95% CI: 1.02-4.05) and being treated for CLL (HR: 1.81; 95% CI: 1.01-3.25) with a higher risk of SADR occurrence [33].

Based on data from a Greek single-center retrospective real-world study including 58 CLL patients (11 first-line, 47 R/R) treated with ibrutinib monotherapy (for a median of 6.6 and 16.3 months, respectively), treatment discontinuation was reported to be associated with AEs (due to atrial fibrillation in 3.5% of patients) in 9% of the first-line and 10.6% of the R/R patients, while it was due to disease progression in 13 (24.5%) patients [34]. These authors concluded that CLL patients had outcomes similar to those of clinical trials if treated homogeneously according to standard guidelines, resulting in fewer unneeded discontinuations and shrinkage of the treatment armamentarium [34]. The superior efficacy of ibrutinib with significantly improved ORR, PFS, and OS compared to ofatumumab in R/R patients or compared to chlorambucil as frontline therapy in TN patients was established in the RESONATE trials, which included extended follow-up analyses [9,13,15,24,35,36,37,38].

Accordingly, our findings support favorable treatment responses and survival outcomes with the use of off-trial ibrutinib, similar to data from multicenter prospective pivotal trials on ibrutinib, despite the fact that patients included in the pivotal clinical trials were often younger, had better ECOG classifications, and presented with milder lymphadenopathy [22,23]. Nonetheless, our findings support the potential roles of poorer ECOG performance status and having been heavily treated before ibrutinib in the likelihood of observing higher treatment discontinuation rates and inferior survival outcome in real-world settings, given the more stringent rules for dose modifications or interruptions and thus higher levels of drug compliance in clinical trials [22].

While del(17p) status had no significant impact on survival outcome in the current study, poorer survival outcome was reported for patients with del(17p) in the 3-year follow-up of a phase 1b-2 multicenter study [37] and in the RESONATE-17 study [39], as well as in a real-life study [23]. However, subgroup analysis of the RESONATE study also showed that the presence of del(17p) was not associated with inferior PFS outcomes with similar ORRs (89% and 91%, respectively) and 18-month PFS rates (71% and 79%, respectively) in patients with del(17p) and those without del(17p) [35]. Likewise, 3-year PFS in ibrutinib-treated CLL patients was reported to be 53% for patients with del(17p), 66% for those with del(11q), and 58% for patients without these abnormalities [40]. In a phase 1b-2 multicenter study of 85 CLL patients, the authors reported ibrutinib to promote durable responses irrespective of the dose, with similar ORRs (71%) in the 420-mg and 840-mg cohorts along with 26-month PFS and OS rates of 75% and 83%, respectively [11]. The authors also noted no significant impact of traditional high-risk prognostic features, including del(17p), on the treatment response rates [11].

Notably, del(17p) has been suggested to be a poor prognostic factor in patients who receive frontline ibrutinib with no negative impact of del(17p) on OS in the R/R setting, while R/R disease, age, performance status, and comorbidities were reported as determinants of poor OS in ibrutinib-treated patients with CLL [41]. Moreover, the frequency of high-risk genomic abnormalities including del(17p) has been suggested to dramatically increase with increasing lines of chemotherapy, and treatment with single-agent ibrutinib earlier in the disease course before the development of these abnormalities has therefore been considered to improve patient outcomes [16].

Indeed, targeted therapies such as ibrutinib are considered to challenge the value of classic prognostic factors defined in the original CLL International Prognostic Index, emphasizing the need for new risk models applicable to CLL patients treated with all currently approved targeted therapies [41,42,43,44].

In the current study, lymphocyte counts increased within the first month of treatment, followed by a gradual decrease starting from the second month. This is consistent with the transient increase in absolute lymphocyte count expected within the first few weeks of ibrutinib therapy, which may persist for several weeks of treatment and does not signify disease progression [24,45]. Nonetheless, some authors reported the association of prolonged treatment-related lymphocytosis with higher likelihood of ibrutinib responders to carry favorable prognostic markers (i.e., del13q and mutated *IGHV*) and a trend toward improved PFS [35,45], while more rapid and more frequent normalization of lymphocyte counts was also reported in patients with unmutated immunoglobulin genes [11].

The safety profile of ibrutinib-treated patients in the current study seems consistent with previous reports, with most AEs being mild to moderate in severity and neutropenia, hypertension, pneumonia, and anemia being the most commonly reported grade 3-4 events [11,15,37,39]. Overall, 176 AEs were reported for 74 (54.4%) of the patients in the current study, with 46 of those 176 AEs being grade 3-4 AEs including pneumonia (n=12), neutropenia (n=11), anemia (n=5), thrombocytopenia (n=5), and fever (n=5) in most cases. The results from the RESONATE trial with up to 5 years of follow-up also showed that the safety profile of ibrutinib over time remains acceptable and manageable and that extended treatment with ibrutinib is tolerable with no long-term safety signals and a reduction in the majority of grade >3 AEs over time, while effective management of AEs during the first year of treatment is considered critical given the highest discontinuation rates within this period [16,24,40].

Consistent with previous real-life data obtained from ibrutinib-treated CLL patients that identified infection as the main cause of death and the common reason for permanent discontinuation of ibrutinib [22,23], our findings revealed sepsis as the leading cause of death among ibrutinib-treated CLL patients. Nonetheless, it should be noted that in a systematic review and meta-analysis of phase III trials with 1227 patients (617 in the ibrutinib arm and 610 in the control arm), the authors concluded that there was no significant increase in the risk of infection associated with ibrutinib in patients with CLL [46].

### Study Limitations

Although the occurrence of atrial fibrillation is generally between 7% and 15% in this age group in real-world analyses, our finding of atrial fibrillation occurrence of only 2% may be explained by the retrospective design of the current study. While the cardiac arrest (14%) and sudden death (3%) rates in our study population indicate a high rate of cardiac death (17%), none of these deaths were related to ibrutinib treatment and they were associated with the high proportion of elderly patients with comorbidities in the study cohort.

## Conclusion

This real-life analysis of CLL patients confirms the favorable efficacy and safety profile of long-term ibrutinib treatment as reported by prospective clinical trials, while emphasizing the potential adverse impact of poorer ECOG performance status, having been heavily treated prior to ibrutinib initiation, and advanced Rai stages but not comorbidity, bulky disease, or del(17p) status on patient compliance, treatment responses, and survival outcomes.

## Figures and Tables

**Table 1:**
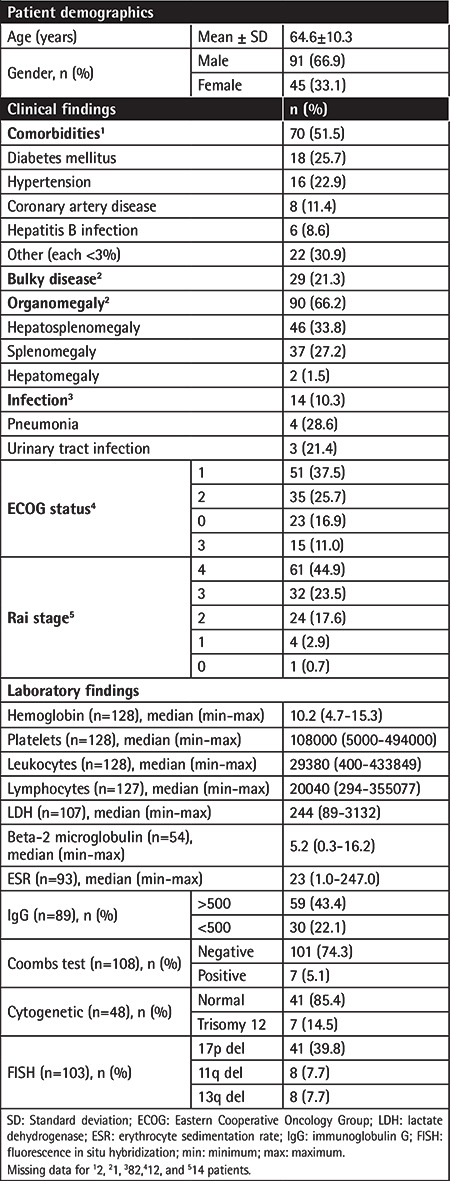
Baseline characteristics of patients.

**Table 2:**
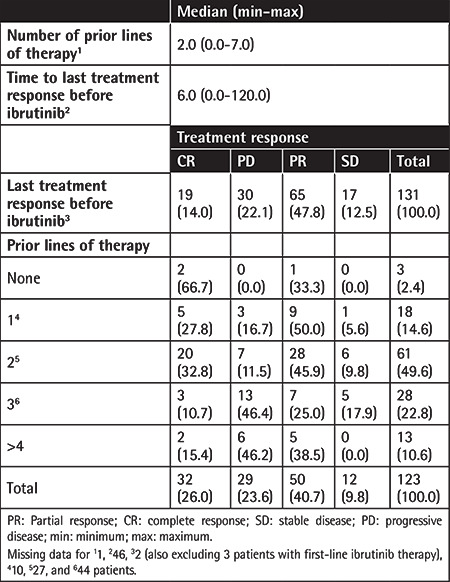
Prior lines of therapy and related treatment responses.

**Table 3:**
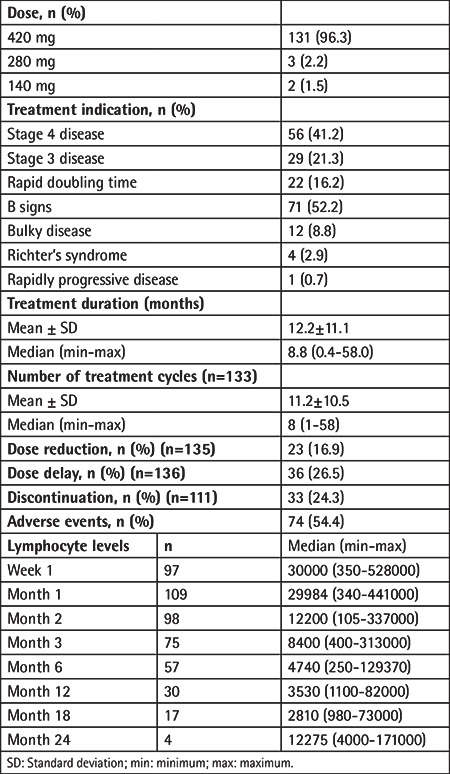
Characteristics of ibrutinib therapy.

**Table 4:**
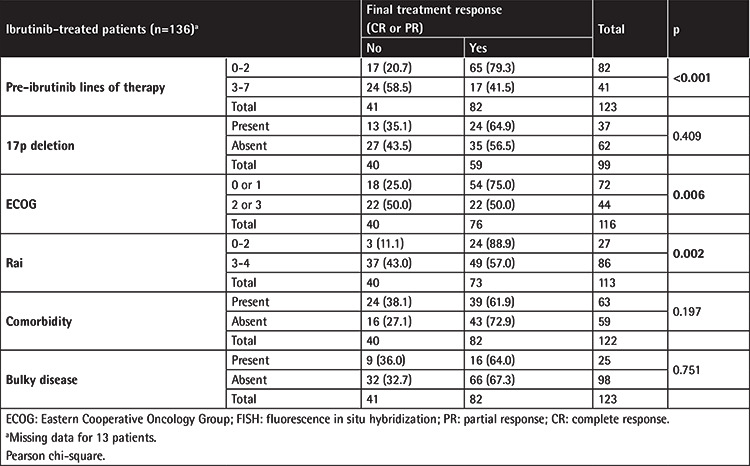
Treatment response with respect to prognostic factors.

**Table 5:**
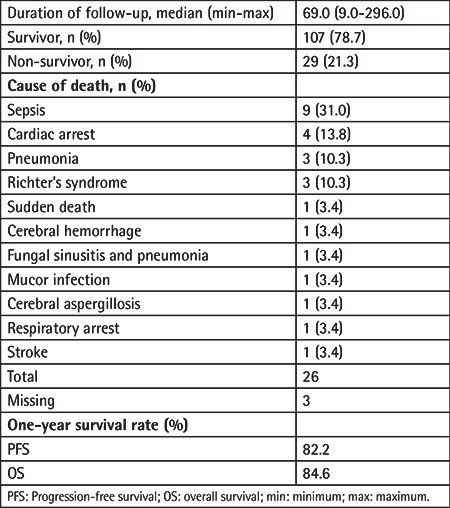
Survival outcome with respect to prognostic factors.

**Table 6:**
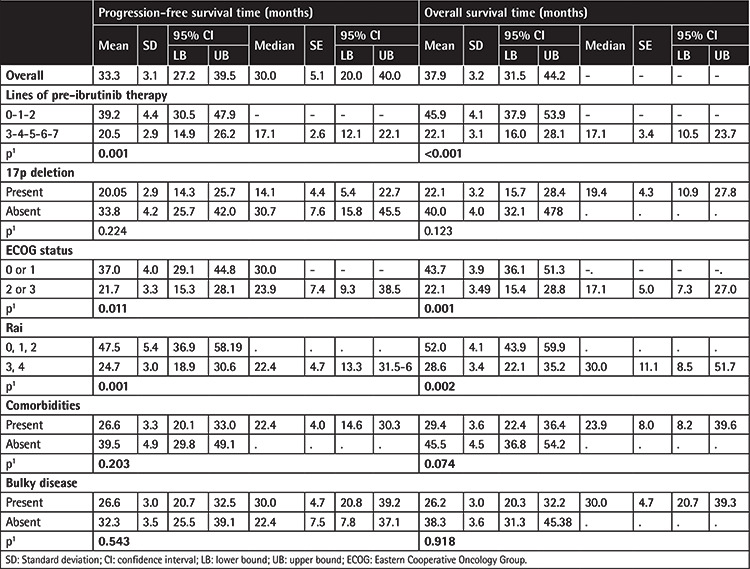
Further analysis of survival outcome with respect to prognostic factors.

**Table 7:**
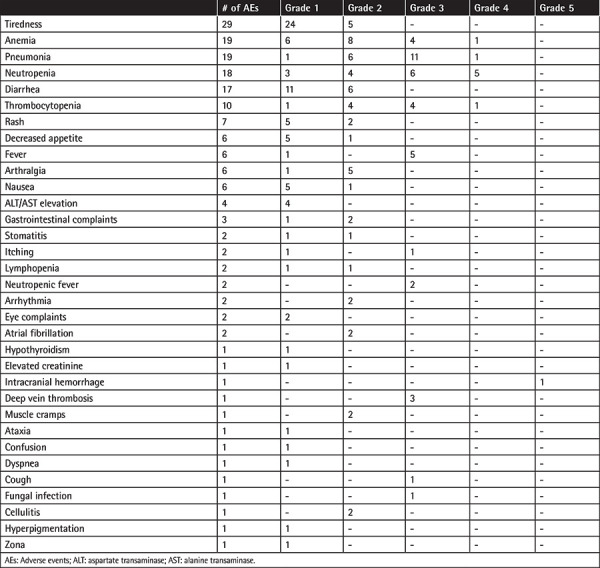
Safety profile.
